# Jejunal Cancer with *WRN* Mutation Identified from Next-Generation Sequencing: A Case Study and Minireview

**DOI:** 10.1155/2014/126924

**Published:** 2014-06-15

**Authors:** Christopher Chang, Her-Shyong Shiah, Nan-Yung Hsu, Hsiu-Ying Huang, Jan-Show Chu, Yun Yen

**Affiliations:** ^1^Department of Molecular Pharmacology, City of Hope National Medical Center and Beckman Research Center, 1500 E Duarte Road, Duarte, CA 91010, USA; ^2^Taipei Medical University, 250 Wu-Hsing Street, Taipei 110, Taiwan

## Abstract

Small bowel cancer is a rare, gastrointestinal cancer originating from the small intestines. Carcinogenesis in the jejunum, the middle segment of the small intestines, occurs less commonly than in the duodenum and ileum. Despite the increasing incidences globally, the cancer is still poorly understood, which includes lack of pathological understanding and etiological reasoning, as it seems to exhibit both similarities and differences with other types of cancers. A 76-year-old Asian man was presented with abdominal pain, which was later attributed to an adenocarcinoma in the jejunum. Initial immunoreactive staining results found no connections to colorectal cancer. The microsatellite instability test was further examined by immunohistochemistry which revealed them to be wild-type. From our exome-capture sequencing results, mutations of *WRN* may be important as they represent the only genetic defect in this jejunal cancer. The patient has since undergone surgical resection of his cancer and is currently being treated with chemotherapy. The pathology, genomic markers, and treatments are described along with literature review.

## 1. Introduction

Small bowel cancer is a relatively rare type of cancer when compared to other gastrointestinal diseases (comprising 2% of all GI cancers), while having glaring similarities to the said malignancies. Typically, the cancer tends to show similarities to malignancies originating from a proximal organ (e.g. stomach and large intestines), explaining the increased rarity of jejunal cancer, as it is surrounded only by its small intestinal counterparts. Factors from neighboring organs have been suspected to affect carcinogenesis in the small bowel, such as bile (and its metabolites) in relation to adenocarcinomas of the duodenum, which again paints the jejunum as an unexpected location of cancer [1]. Because few case studies and literature reviews exist for jejunal cancer, we will largely review small bowel cancer as a whole.

**Figure 1:**
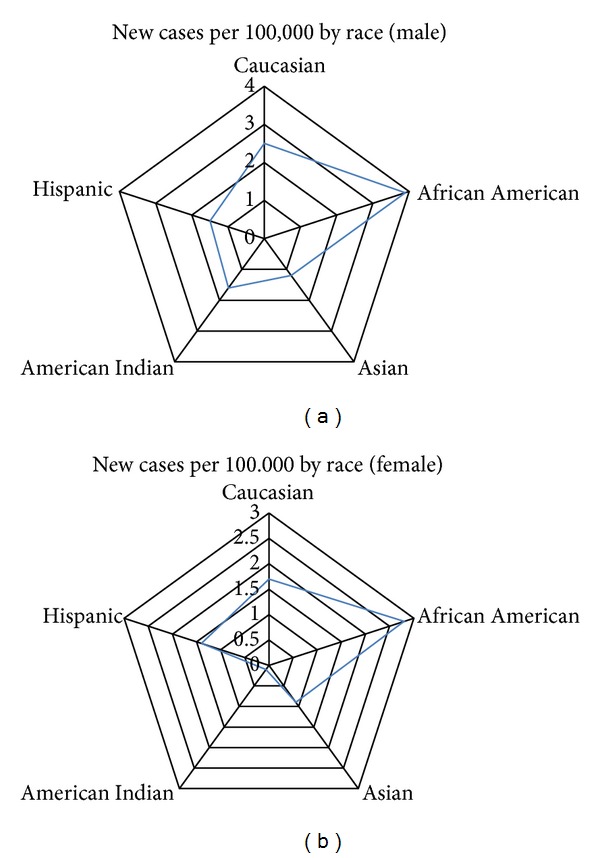
Graphs showing the incidence rate split between gender and race in the United States.

In the United States, African Americans are twice as likely to be diagnosed with small bowel cancer as compared to Caucasians. Also, men of all races have a higher incidence rate for this cancer than women across the races (roughly 1.5 times higher) (Figure 1). Like many other types of cancer, genetic and environmental risks represent susceptibility factors to small intestine cancer. Although the tumor can be diagnosed with endoscopic tools, preventative care is nearly impossible due to the lack of pathological knowledge in this field due to small sample sizes and few defining symptoms during the early stages.

Though the limitations in predicting small bowel cancer preempt preventative medicine, chemotherapy can be used to slow the tumor growth albeit to a lesser extent; for example, chemotherapeutic drugs for adenocarcinomas in the stomach have been found to be less effective on adenocarcinomas in the small intestines. Surgical treatment remains the most effective way to treat small bowel cancer, increasing the survivability considerably and eliminating obstructions.

## 2. Case Study of Patient

An Asian businessman, aged 76, was initially discovered to have adenocarcinoma fragments in his colonic mucosal tissue. These carcinoma cells were stained and found to be immunoreactive for CKAE1/AE3 and CK7 while nonreactive for CK20 and LCA. This indicates that the tumor was of epithelial nature and was possibly derived from cells in the bile duct, esophagus, lung, or mesothelium. More importantly, these results (CK7+/CK20−) indicated that the malignancy did not originate from colon, while the negative LCA suggest that the tumor did not derive from hematopoietic cells (Table 1). The histologic and immunophenotypic findings were more indicative of invasive adenocarcinomas due to differences from colonic adenocarcinomas and subsequent CT scan suggested that they originated from the adjacent small intestines, specifically, the jejunum.

During the surgery, it was concluded that the tumor of jejunum as the primary site, which directly invaded into the hepatic flexure of colon, resulted in a fistula formation between jejunum and colon. Four of fifteen mesenteric and mesocolic lymph node samples had metastatic adenocarcinoma growth, as did the greater omentum. Proximal and distal cuts of the small intestine and colon, respectively, were free of tumors. The final diagnoses of the malignancy reached the pT4N2M1 status. More recent CT scans revealed that the disease has metastasized to the lungs and peritoneum.

The KRAS and EGFR mutation tests were performed to detect variations in codons 12/13 and exons 18–21, respectively. Mutations in codons 12 and 13 of KRAS have been correlated with poor response to anti-EGFR monoclonal antibody therapies in patients with colorectal cancer [2, 3]. No mutations were found in this patient (Table 2).

The male patient has a history of hypertension, carotid artery stenosis, and fatty liver. He is a carrier of the hepatitis B virus and had peptic ulcers 40+ years ago. His family history revealed that his older brother died of colon cancer at the age of 49. There were no abnormalities during his physical testing.

The patient's medication consists of Ferall soft capsules (for his anemia), Cozaar (for his hypertension), Plavix (to prevent stroke and heart disease), Xatral (to alleviate urination), Baraclude (to treat chronic hepatitis B infections), and proton pumps inhibitor for his non-*H. pylori* chronic peptic ulcer disease. Chemotherapy of FOLFOX followed by FOLFIRI was further given in treating his jejunum cancer.

## 3. Mini Review

### 3.1. Background

Small bowel cancer is a highly variable cancer (typewise) depending on its location. While 98% of all small bowel cancers consist of adenocarcinomas, carcinoid tumors, lymphomas, and sarcomas/gastrointestinal stromal tumors (GISTs) [4], their classifications and properties reflect nearby organs. GISTs are commonly found in the stomach and are infrequent in the colon; GISTs located in the small intestine are typically found in the duodenum as it is the closest to the stomach. 30% of all GISTs occur in the duodenum, with most of the remaining percentage in the stomach [5]. Likewise, adenocarcinomas found in the jejunum and ileum closely resemble those from colorectal cancer while adenocarcinomas in the duodenum are generally more similar to those in the stomach.

Only a small percentage of all gastrointestinal cancers occur in small bowel because of particular enzymes found along its tract that inactivate carcinogens. One such enzyme is benzopyrene hydrolase, which targets the potent carcinogen benzopyrene. The enzyme is especially helpful considering the high prevalence of benzopyrene, for example, cigarette smoke, burnt foods, and forest fires [6].

The prognoses vary depending on the different types of small bowel cancer as well as the stages. The overall five-year survival rate for adenocarcinoma patients is 15–30%. It rises to 40–60% for those with resectable lesion with little chance for survival for the metastasized. For the latter, surgery may be performed to relieve symptoms caused by tumor obstruction. While the mean age of acquisition is 60 years, a study of 38 patients has determined that age is not a factor for survivability [7]. A delay in diagnoses often proves fatal due to lymph node metastases. An early diagnosis and removal of the tumor can greatly improve long-term survivability.

### 3.2. Epidemiology

Although small bowel cancer is the rarest GI cancer, its increasing incidence is alarming. In 2001, there were approximately 4,800 new cases in the United States with 1,200 deaths [8]. In 2008, more than 6,110 new cases occurred [9]. In 2013, an estimated 8,800 adults were diagnosed with small bowel cancer [10]. Fortunately, the number of deaths has remained constant at 1,200 deaths per year, but its increased incidence should not be overlooked. Regardless of whether it resulted from an increased awareness or represents an escalation, proper measures should be taken to prevent further occurrences.

**Figure 2:**
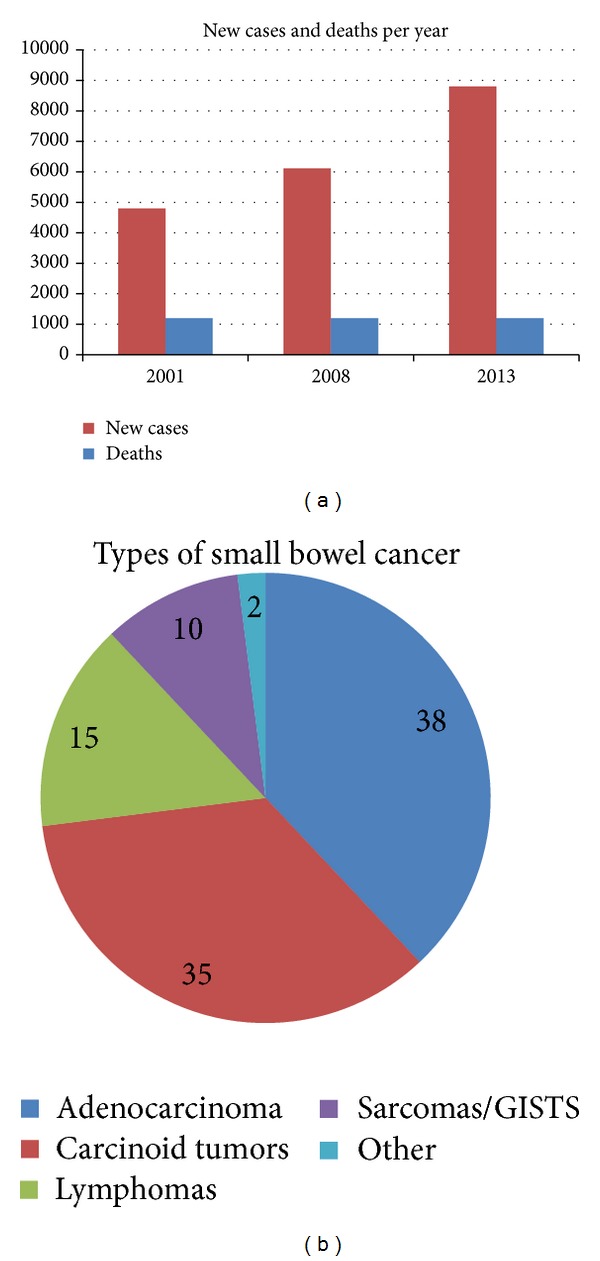
Charts showing epidemiological data for the US population.

Of the four major types of small bowel cancer, adenocarcinomas are the most common in the United States, constituting approximately 40% of the diagnosed. They originate from gland cells alongside the epithelial lining of the small intestine and can obstruct digestion if greatly enlarged. Whereas it poses a greater concern for the US and other Western countries' residents, the rest of the world sees fewer cases of adenocarcinoma with small bowel cancer, particularly in parts of the eastern hemisphere and less industrialized countries. Rather, small intestinal cancers in the east are predominately lymphomas. Of the adenocarcinoma cases in the United States, a greater percentage occurs in the duodenal region of the small intestine than the latter two sections—approximately 50% in the duodenum, 30% in the jejunum, and 20% in the ileum [11]. The reason for the discrepancy is unknown (Figure 2).

### 3.3. Etiology

Genetic, environmental, and medical conditions all represent susceptibility factors that predispose to small bowel cancer. Genetic risks include familial adenomatous polyposis (FAP), hereditary nonpolyposis colorectal cancer (HNPCC), and Peutz-Jegher syndrome (PJS). FAP is a hereditary condition in which the epithelium of the large intestine is prone to numerous polyp growth. Although polyps are benign, the continual generation of polyps increases the risk of mutations, thus frequently leading to colon cancer. Its hereditary pattern varies depending on the target gene mutation (it can be autosomal dominant or recessive) [12]. HNPCC is an autosomal dominant disease that predisposes to many types of cancer mainly not only in the colon but also in small intestine and several other organs. PJS, like FAP, populates the colon with hamartomatous polyps and is an autosomal dominant disease. Other symptoms of PJS include hyperpigmented macules on the oral mucosa.

Whether environmental elements cause small bowel cancer is unknown. In 1986, it was found that alcohol consumption and tobacco use did not increase the risk for the cancer though certain diets did [13]. Eating red meat at least once a week and smoked foods more than once a month increased two- to threefold the risk for small bowel cancer [13]. In 2012, a positive correlation for added fructose and small intestinal cancer was identified [14]. Industrialized countries have a higher occurrence rate for small bowel cancer though the underlying cause is unclear [1].

Diseases like Crohn's disease and Celiac disease may represent a subset of preexisting condition for small bowel cancer [15]. Crohn's disease occurs in individuals interacting with certain environmental and immunological factors as it was originally thought to induce immune system to react against the gastrointestinal tract for microbial antigens, leading to chronic inflammation. Yet, many now currently consider that the inflammation is caused by immune deficiency. Genetics and smoking tobacco can increase the likelihood for contracting this disease. Unlike Crohn's disease, Celiac disease is an autoimmune disorder that specifically targets the small intestine, especially the genetically predisposed individuals. Caused by prolamin, a gluten protein, this disease impairs the function of the small intestine to absorb nutrients, leading to malnutrition complications, such as vitamin deficiency [16].

### 3.4. Diagnosis

Symptoms of small bowel cancer usually arise due to obstructions in the small intestine caused by the enlarged tumor mass. Similar to irritable bowel syndrome (IBS), peptic ulcer disease (PUD), and colon cancer, these symptoms can include abdominal discomfort/pain, physical deformation of the abdomen, loss of appetite, weight loss, weakness, nausea, vomiting, jaundice, and even melena. Small bowel cancer can be distinguished from IBS/PUD by recording the duration and recurrence of the symptoms. Small bowel cancer symptoms can last for several weeks or longer while IBS/PUD usually resolves within a few weeks. By the time these symptoms are detected, the tumor may have progressed to late stages and/or metastasized. A key distinction between small bowel cancer and colon cancer is the color of the stool. Colon cancer can lead to either melena or hematochezia, whereas small bowel cancer primarily presents melena.

Several techniques exist for examination when small bowel cancer is suspected. First, a blood sample from the patient can be tested for a complete blood count (CBC) to see if anemic (small intestine cancer often causes low red blood cell count). It can be further tested for liver disease and high levels of certain proteins that may be caused by aggressive tumor growth. Fecal blood tests may be performed if melena is suspected. With enough evidence, the next step can consist of imaging tests, endoscopies, and/or biopsies. While imaging tests are noninvasive, there can be small but deleterious effects caused by radiation (barium X-rays) or radioactive substances (PET scans), for instance. Although CT scans are safer alternatives, they are not as powerful as the other two tests.

Endoscopy is mainly utilized for physically viewing (with a camera) the internal linings of a patient with GI tract complications. Double-balloon enteroscopy (DBE) represents a recent form of endoscopy proven to be effective for small bowel cancer [17]. Unlike traditional endoscopy, DBE uses a special endoscope made up of two tubes with one inside the other to reach deep enough into the small intestines. An intuitive inflate-and-anchor rhythm is used to inflate the balloon at the tip of the tube and visualize one foot of the small intestine before deflating and moving on to the next section. The point of entry for the tubes can be either through the mouth or through the anus, depending on how far the target region is. Accuracy of DBE for adenocarcinomas is >90% in most cases, with an even higher success rate for other types of small bowel cancer [18]. Should visual analysis of the tumor be difficult, DBE can also be used as a tool for sampling and biopsy of the small intestinal tissue in place of stents or dilatations.

### 3.5. Treatment

Because of the relatively few cases of small bowel cancer, few defining medical treatments exist other than the conventional therapy for similar cancers such as those found in the stomach and colon. Even then, conventional chemotherapeutic treatments on adenocarcinomas of the small bowel produce less favorable results than they do with the stomach [19].

Surgery (when possible) is the preferred route in increasing a patient's 5-year survival rate. For adenocarcinomas, radical surgery can be performed on resectable tumors. It involves removing the blood supply and lymphatic system supplying the organ. For unresectable tumors (tumors that are extensive and/or have metastasized to multiple distant organs), a surgical bypass is the only other solution to avoid further obstructions. Laparoscopic surgery, a minimally invasive surgery, can be an alternate choice when tumor sizes are smaller than 10 cm in diameter. In fact, it is the preferred option for patients because of the favorable postsurgical outcomes, without loss in efficacy as compared to open surgery [20].

## 4. Discussion

Despite their similarities, the traits of small bowel cancer vary from other gastrointestinal cancers. Though many risk factors for small bowel cancer like Peutz-Jeghers syndrome (PJS) and hereditary nonpolyposis colorectal cancer (HNPCC) are analogous to colon cancer, some patients may not present any resembling evidence.

The aforementioned Asian man went through a series of tests, including microsatellite instability staining and tumor mutation tests, to find attributes of colorectal cancer. A targeted gene sequencing test (next-generation sequencing (NGS)) for 88 genes related to colon cancer (see Appendix) found mutations in two different genes: the* PMS2* (p.Arg20Gln) and the* WRN* (p.Leu628Val) genes (for more information, see Table 3). In humans, the* PMS2* gene located in chromosome 7 encodes the mismatch repair endonuclease PMS2 that functions to correct errors found during DNA replication.* WRN* encodes the Werner protein, a DNA helicase used for DNA repair, recombination, and replication.

Mutations in* PMS2* lead to Lynch syndrome (LS), an autosomal genetic disease with a high risk of colon cancer [21]. Like HNPCC, LS may increase risks for cancer in the ovaries, stomach, small intestine, upper urinary tract, and the brain [22]. Mutations in* WRN* cause Werner syndrome (WS), an autosomal recessive disease; it may prevent the helicase from entering the cell nucleus, typically leading to premature aging symptoms and earlier onset of cancer [23]. With respect to colorectal cancer, hypermethylation of the* WRN* promoter is commonly found, reducing its expression [24]. With the* PMS2* and* WRN* results, a pathological connection can be found between the patient's jejunal cancer and colorectal cancer.

The staining of microsatellite instability (MSI) proteins MLH1, MSH2, MSH6, and PMS2 on the patient yielded wild-type results. These proteins, associated with Turcot syndrome (a mismatch repair cancer syndrome) when defected, are mainly involved in DNA mismatch repair. Mutations in the genes encoding these proteins are linked to hereditary nonpolyposis colorectal cancer (HNPCC). Turcot syndrome, caused by its biallelic mutations, is mainly characterized by tumor growth in the primary nervous system, leading to neurological symptoms such as muscle spasms.

MSI's 5 selective markers are used to detect instability for microsatellites. Detection of as few as 2 markers may be considered a positive result. For this patient, the positive staining result meant that the proteins function normally in this adenocarcinoma of the jejunum (Table 4). Although seemingly contradictory to the DNA sequencing results that detected PMS2 mutations, the positive MSI result for the normal PMS2 protein may indicate that they represent silent mutations. Thus, the significance of* PMS2* can be downplayed due to the MSI results.

Wheeler et al. [25] as well as others understood the complications with small bowel cancer. In their 2001 study, Wheeler and his colleagues sought to define the genetic basis of adenocarcinomas of the small intestine. Twenty-one participants with a median age of 64 years, all of whom had nonfamilial adenocarcinomas in the small intestine, volunteered to undergo a series of genetic and protein tests, which include the MLH1 and MSH2 proteins and* APC* gene. Similar to our results, the proteins were all found to be normal. Mutations in the APC gene, which is uncommonly associated with colorectal cancer and Turcot syndrome, were not discovered either, despite murine studies by Arber et al. [26] claiming modifications in the gene to be highly correlated with small bowel tumorigenesis in mice.

The death of his brother due to colon cancer at the age of 49 may suggest that the 76-year-old man was possibly genetically predisposed. With that, our target for analysis of our patient data will be on the outlier not found in others' studies:* WRN*. Of the mutations found in the gene sequencing tests, only* WRN* may relate to jejunal cancer. Little information exists in the literature relating small bowel cancer and the* WRN *gene. Although infrequent,* WRN* defects are associated with gastric cancer roughly 25% of the time [27], while it is considered a top ranked gene associated with advanced colorectal cancer. The effects of* WRN *mutation can vary depending on its location on the gene, such that an irregularity in the gene (e.g., Gly574Arg) may not necessarily cause the usual symptoms of premature aging. This implies that the helicase deficiency from* WRN* mutations may not necessarily be the cause for the short stature of Werner syndrome patients.

Because* WRN* mutations lead to double-stranded DNA damage repair entropy, the increased genomic instability is a cause for concern for any sort of cancer. Saha et al. [28] ventured into possible treatments which can be used to decrease the damage caused by mutations in* WRN* and found rapamycin as a potential therapeutic solution. Rapamycin, also known as sirolimus, is an immunosuppressant drug that plays a role in the mammalian target of rapamycin (mTOR) pathway, which is known to control cell growth, proliferation, and survival. Dysregulation of the pathway typically increases the risk for cancer.

Rapamycin specifically inhibits the mTOR complex 1 (mTORC1), an important protein complex that controls protein synthesis by activating translation. Using* WRN* knockdown fibroblasts, Saha et al. initially discovered rapamycin treatment to increase autophagy in human telomerase- (hTERT-) immortalized cells. A problem with Werner syndrome patients' cells is that, because of the inability for the* WRN* helicase to function normally, a buildup of insoluble proteins aggregates occurs with increasing oxidative damage to the cell. Saha et al. observed an elimination of these protein aggregates via autophagy when treated short-term with rapamycin. In the long-term treatment, DNA damage accumulation was reduced, growth rates were improved, and nuclear morphology was improved with autophagy markers returning back to baseline levels, probably due to the prior clearance of irregular proteins [28].

Because of our limited knowledge, small bowel cancer is still treated as if it were gastric or colorectal cancer despite the substandard efficacy. Molecular pathogenesis examining pathways yielded little success. Nonetheless, it may lead to finding the optimal targets for treatment. Our genetic tests on genes such as* WRN* lead us to recognize possible pathologies to jejunal cancer. Whether it represents an isolated case, further examination of the* WRN *gene may provide additional insight for the scientific community.

## Figures and Tables

**Table 1:** Immunohistochemistry staining results of the Asian patient. A positive result for immunoreactivity staining indicates reactivity.

Immunohistochemistry staining profile	Test results for patient
(i) CKAE1/AE3	+
(ii) CK7	+
(iii) CK20	−
(iv) LCA	−

**Table 2:** Mutation test results of the Asian patient. A positive result for mutation tests indicates mutations in the specified regions.

KRAS and EGFR mutation tests	Test results for patient
(i) KRAS codons 12/13	−
(ii) EGFR exon 19	−

**Table 3:** Gene sequencing results for the Asian patient. Mutation information is provided accordingly.

Genomic profiling	Variant	SNP ID	Zygosity	References
*PMS2* mutation	NM_000535.5:c.59G>A, p.Arg20Gln	rs10254120	Heterozygous	[29, 30]
*WRN* mutation	NM_000553.4:c.1882C>G, p.Leu628Val	rs77969734	Heterozygous	[31]

Next-generation sequencing (NGS): genomic DNA was isolated from submitted specimens. DNA was barcoded and enriched for the coding exons of queried genes using hybrid capture technology. The prepared DNA library was then sequenced using a next generation sequencing platform. Variants were detected in regions of 20x coverage where 20% of alleles were variant. 93% of targeted regions had at least 20x coverage. The remaining regions did not have 20x coverage and were not evaluated. The following variants were filtered: those with dsSNP minor allele frequency >1%, those noted as nonpathogenic in dsSNP, and those not resulting in amino acid alterations when no obvious splicing effect was anticipated. The remaining variants were interpreted manually.

**Table 4:** MSI test results for the Asian patient. A positive MSI indicates normal proteins.

Microsatellite instability staining	Test results for patient
(i) MLH1	+
(ii) MSH2 and MSH6	+
(iii) PMS2	+

## Appendix

Genes queried: APC, ATM, ATR, AXIN2, BAP1, BARD1, BLM, BMPR1A, BRCA1, BRCA2, BRIP1, BUB1B, CDH1, CKD4, CKDN2A, CHEK2, CTNNB1, CYLD, DDB2, DICER1, EPCAM, ERCC2, ERCC3, ERCC4, ERCC5, EXO1, EXT1, EXT2, FANCA, FANCC, FANCD2, FANCF, FANCG, FLCN, GPC3, HRAS, KIT, MC1R, MEN1, MITF, MLH1, MPL, MRE11A, MSH2, MSH3, MSH6, MUTYH, NBN, NF1, NF2, PALB2, PDGFRA, PMS2, PMS2, PRKAR1A, PRKDC, PRSS1, PTCH1, PTEN, PTPN11, RAD50, RAD51, RAD51C, RAD51D, RB1, RBBP8, RET, ROBO2, SBDS, SDHB, SDHC, SDHD, SMAD4, SMARCB1, STK11, SUFU, TERT, TP53, TSC1, TSC2, TSHR, TYR, VHL, WRN, WT1, XPA, XPC, XRCC3.

## References

[1] Neugut AI, Jacobson JS, Suh S, Mukherjee R, Arber N (1998). The epidemiology of cancer of the small bowel. *Cancer Epidemiology Biomarkers and Prevention*.

[2] Arai M, Shimizu S, Imai Y (1997). Mutations of the Ki-ras, p53 and APC genes in adenocarcinomas of the human small intestine. *International Journal of Cancer*.

[3] de Biase D, Visani M, Malapelle U (2013). Next-generation sequencing of lung cancer EGFR exons 18-21 allows effective molecular diagnosis of small routine samples (cytology and biopsy). *PLoS ONE*.

[4] Somasundar P, Fisichella P, Espat N Malignant Neoplasms of the Small Intestine.

[5] Emory TS, Sobin LH, Lukes L, Lee DH, O’Leary TJ (1999). Prognosis of gastrointestinal smooth-muscle (stromal) tumors: dependence on anatomic site. *American Journal of Surgical Pathology*.

[6] Izbicki JR, Nagelschmidt M, Dornschneider G, Kusche J, Schmitz R (1987). Proteolytic enzymes as new tumor markers in chemical carcinogenesis of intestinal tumors. *Cancer Detection and Prevention*.

[7] Bauer RL, Palmer ML, Bauer AM, Nava HR, Douglass HO (1994). Adenocarcinoma of the small intestine: 21-year review of diagnosis, treatment, and prognosis. *Annals of Surgical Oncology*.

[8] Neugut AI, Marvin MR, Chabot JA, Holzheimer RG, Mannick JA (2001). Adenocarcinoma of the small bowel. *Surgical Treatment: Evidence-Based and Problem-Oriented*.

[9] Overman MJ (2009). Recent advances in the management of adenocarcinoma of the small intestine. *Gastrointestinal Cancer Research*.

[10] *Surveillance, Epidemiology, and End Results Program: Turning Cancer Data Into Discovery*.

[11] American Cancer Society Small Intestine Cancer. http://www.cancer.org/acs/groups/cid/documents/webcontent/003140-pdf.pdf.

[12] Ibrahim A, Barnes DR, Dunlop J, Barrowdale D, Antoniou AC, Berg JN (2014). Attenuated familial adenomatous polyposis manifests as autosomal dominant late-onset colorectal cancer. *European Journal of Human Genetics*.

[13] Chow W-H, Linet MS, McLaughlin JK, Hsing AW, Co Chien HT, Blot WJ (1993). Risk factors for small intestine cancer. *Cancer Causes and Control*.

[14] Tasevska N, Jiao L, Cross AJ (2012). Sugars in diet and risk of cancer in the NIH-AARP diet and health study. *International Journal of Cancer*.

[15] Potter DD, Murray JA, Donohue JH (2004). The role of defective mismatch repair in small bowel adenocarcinoma in celiac disease. *Cancer Research*.

[16] di Sabatino A, Corazza GR (2009). Coeliac disease. *The Lancet*.

[17] Lee S-Y, Tomoyoshi S, Haga K (2012). Multiple carcinoid tumors of the small intestine preoperatively diagnosed by double-balloon endoscopy. *Medical Science Monitor*.

[18] Chen WG, Shan GD, Zhang H (2013). Double-balloon enteroscopy in small bowel tumors: a Chinese single-center study. *World Journal of Gastroenterology*.

[19] Jigyasu D, Bedikian AY, Stroehlein JR (1984). Chemotherapy for primary adenocarcinoma of the small bowel. *Cancer*.

[20] Ihn K, Hyung WJ, Kim H-I (2012). Treatment results of small intestinal gastrointestinal stromal tumors less than 10 cm in diameter: a comparison between laparoscopy and open surgery. *Journal of Gastric Cancer*.

[21] Gulati S, Gustafson S, Daw HA (2011). Lynch syndrome associated with PMS2 mutation: understanding current concepts. *Gastrointestinal Cancer Research*.

[22] Kastrinos F, Mukherjee B, Tayob N (2009). Risk of pancreatic cancer in families with Lynch syndrome. *Journal of the American Medical Association*.

[23] Lauper JM, Krause A, Vaughan TL, Monnat RJ (2013). Spectrum and risk of neoplasia in Werner syndrome: a systematic review. *PLoS ONE*.

[24] Kawasaki T, Ohnishi M, Suemoto Y (2008). WRN promoter methylation possibly connects mucinous differentiation, microsatellite instability and CpG island methylator phenotype in colorectal cancer. *Modern Pathology*.

[25] Wheeler JMD, Warren BF, Mortensen NJM (2002). An insight into the genetic pathway of adenocarcinoma of the small intestine. *Gut*.

[26] Arber N, Neugut AI, Bernard Weinstein I, Holt P (1997). Molecular genetics of small bowel cancer. *Cancer Epidemiology Biomarkers and Prevention*.

[27] Wang L, Shen J, Meng LJ, Fan WF, Wang J, Liu BR (2013). Correlation between the methylation of SULF2 and WRN promoter and chemosensitivity to irinotecan in gastric cancer. *Zhonghua Zhong Liu Za Zhi*.

[28] Saha B, Cypro A, Martin GM, Oshima J (2014). Rapamycin decreases DNA damage accumulation and enhances cell growth of WRN-deficient human fibroblasts. *Aging Cell*.

[29] Johnston JJ, Rubinstein WS, Facio FM (2012). Secondary variants in individuals undergoing exome sequencing: screening of 572 individuals identifies high-penetrance mutations in cancer-susceptibility genes. *American Journal of Human Genetics*.

[30] Pastrello C, Pin E, Marroni F (2011). Integrated analysis of unclassified variants in mismatch repair genes. *Genetics in Medicine*.

[31] Hsu JJ, Kamath-Loeb AS, Glick E (2010). Werner syndrome gene variants in human sarcomas. *Molecular Carcinogenesis*.

